# Exploiting the Natural Diversity of RhlA Acyltransferases for the Synthesis of the Rhamnolipid Precursor 3-(3-Hydroxyalkanoyloxy)Alkanoic Acid

**DOI:** 10.1128/AEM.02317-19

**Published:** 2020-03-02

**Authors:** Andrea Germer, Till Tiso, Conrad Müller, Beate Behrens, Christian Vosse, Karen Scholz, Matti Froning, Heiko Hayen, Lars M. Blank

**Affiliations:** a RWTH Aachen University, iAMB (Institute of Applied Microbiology, ABBt), Aachen Biology and Biotechnology, Aachen, Germany; b University of Münster, Institute of Inorganic and Analytical Chemistry, Münster, Germany; University of Bayreuth

**Keywords:** RhlA, HAA, rhamnolipids, glycolipids, chain length, 3-(3-hydroxyalkanoyloxy)alkanoic acid

## Abstract

While rhamnolipids of the Pseudomonas aeruginosa type are commercially available, the natural diversity of rhamnolipids and their origin have barely been investigated. Here, we collected known and identified new *rhlA* genes encoding the acyltransferase responsible for the synthesis of the lipophilic rhamnolipid precursor 3-(3-hydroxyalkanoyloxy)alkanoic acid (HAA). Generally, all homologs were found in *Betaproteobacteria* and *Gammaproteobacteria*. A likely horizontal gene transfer event into *Actinobacteria* is the only identified exception. The phylogeny of the RhlA homologs from *Pseudomonas* and *Burkholderia* species is consistent with the organism phylogeny, and genes involved in rhamnolipid synthesis are located in operons. In contrast, RhlA homologs from the *Enterobacterales* do not follow the organisms’ phylogeny but form their own branch. Furthermore, in many *Enterobacterales* and *Halomonas* from the *Oceanospirillales*, an isolated *rhlA* homolog can be found in the genome. The RhlAs from Pseudomonas aeruginosa PA01, Pseudomonas fluorescens LMG 05825, Pantoea ananatis LMG 20103, Burkholderia plantarii PG1, Burkholderia ambifaria LMG 19182, *Halomonas* sp. strain R57-5, Dickeya dadantii Ech586, and Serratia plymuthica PRI-2C were expressed in Escherichia coli and tested for HAA production. Indeed, except for the *Serratia* RhlA, HAAs were produced with the engineered strains. A detailed analysis of the produced HAA congeners by high-performance liquid chromatography coupled to tandem mass spectrometry (HPLC-MS/MS) highlights the congener specificity of the RhlA proteins. The congener length varies from 4 to 18 carbon atoms, with the main congeners consisting of different combinations of saturated or monounsaturated C_10_, C_12_, and C_14_ fatty acids. The results are discussed in the context of the phylogeny of this unusual enzymatic activity.

**IMPORTANCE** The RhlA specificity explains the observed differences in 3-(3-hydroxyalkanoyloxy)alkanoic acid (HAA) congeners. Whole-cell catalysts can now be designed for the synthesis of different congener mixtures of HAAs and rhamnolipids, thereby contributing to the envisaged synthesis of designer HAAs.

## Abstract (precis)

The RhlA specificity explains the observed differences in 3-(3-hydroxyalkanoyloxy)alkanoic acid (HAA) congeners. Whole-cell catalysts can now be designed for the synthesis of different congener mixtures of HAAs and rhamnolipids, thereby contributing to the envisaged synthesis of designer HAAs.

## INTRODUCTION

Surfactants are amphiphilic molecules that reduce surface and interfacial tensions, which allows them to accumulate at interfaces and form emulsions. These properties are of industrial interest and are exploited in multiple applications in such different fields as pharmaceuticals, agriculture, food, detergents, and cosmetics (1–3). Biosurfactants are surfactants of biological origin and are a promising alternative to synthetic surfactants, as they are nontoxic, biodegradable, and produced from renewable feedstocks. Their application window is extensive, as they might be effective in environments with extreme pH, temperature, or salinity (4–6).

**FIG 1:**
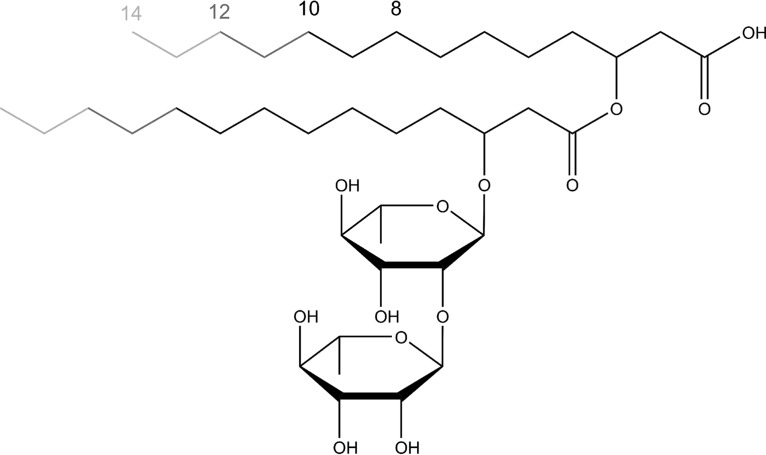
Molecular structure of a rhamnolipid molecule. The chain lengths of the hydroxy fatty acids vary, resulting in different congeners. The main congener produced by P. aeruginosa contains 10 carbon atoms in both hydroxy fatty acid derivatives. Without the two rhamnose units, the molecule is a 3-(3-hydroxyalkanoyloxy)alkanoic acid (HAA). The synthesis of an HAA molecule is catalyzed by RhlA, which fuses two hydroxy fatty acids. RhlB links an activated dTDP-rhamnose to an HAA, resulting in a mono-rhamnolipid, which is the substrate that is transformed by RhlC, the second rhamnosyltransferase, into a di-rhamnolipid.

The biosurfactant 3-(3-hydroxyalkanoyloxy)alkanoic acid (HAA) is the hydrophobic moiety of rhamnolipids and most often consists of two hydroxy fatty acids linked by an ester bond (4, 7–10) (Fig. 1). Indeed, HAAs are not reported as typical products of microorganisms but, rather, were reported in trace amounts during rhamnolipid formation (11).

**FIG 2:**
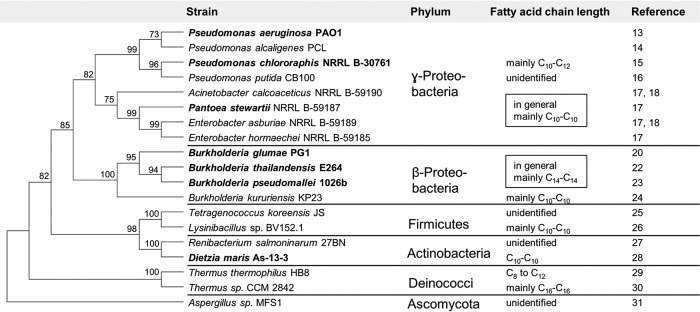
Phylogeny of published rhamnolipid producers based on 16S or 18S rRNA gene sequences. Strains in which *rhl* genes for rhamnolipid synthesis have been sequenced are marked in bold. The rRNA gene sequence of a reference strain was chosen to represent unsequenced rhamnolipid producers. The tree was constructed using the neighbor-joining method in MEGA7 with default settings. The numbers indicate bootstrap results.

Jarvis and Johnson identified rhamnolipids in 1949 (12). Since then, these biosurfactants have been produced with different *Pseudomonas* species. Pseudomonas aeruginosa, a representative of the phylum *Gammaproteobacteria*, is one of the main producers in academia and industry. In the course of the past 2 decades, powerful analytical equipment such as Fourier transform infrared (FT-IR) spectroscopy and high-performance liquid chromatography coupled to tandem mass spectrometry (HPLC-MS/MS) has enabled researchers to detect and verify the presence of rhamnolipids in culture supernatants. Rhamnolipid producers have been reported in the following phyla: *Betaproteobacteria* and *Gammaproteobacteria* (13–24), *Firmicutes* (25, 26), *Actinobacteria* (27, 28), *Deinococcus-Thermus* (29, 30), and *Ascomycota* (31) (Fig. 2).

The carbon chain lengths of HAAs determine their physical properties, such as their abilities to foam and emulsify, and their critical micelle concentration (CMC). Their chain lengths are strongly hinted to be determined by RhlA, an acyltransferase containing an α-/β-hydrolase domain that catalyzes the esterification of two activated hydroxy fatty acids to HAA (32). In *in situ* experiments, it has been shown that acyl-carrier protein (ACP)-activated hydroxy fatty acids are the preferred substrate for RhlA (8), while it has been shown *in vivo* in P. aeruginosa that CoA-activated hydroxyl fatty acids are incorporated preferably into the HAA molecule (33). Within the *Gammaproteobacteria*, *Pseudomonas*, Acinetobacter, *Enterobacter* (17, 18), and *Pantoea* (34) species produce mono- or diglycolipids. Their chain lengths vary, while the most common HAAs have 10 carbon atoms in both hydroxy fatty acids and are thus denoted C_10_-C_10_. In contrast, representatives of the *Betaproteobacteria*, namely, *Burkholderia* species, predominantly produce HAAs with chain lengths of 14 carbon atoms (Fig. 2). A few species do not follow this general categorization. Pseudomonas chlororaphis, e.g., produces rhamnolipids with one fatty acid chain of 10 carbon atoms and one of 12, resulting in the designation Rha-C_10_-C_12_ when these chains are fully saturated and Rha-C_10_-C_12:1_ when the C_12_ chain is unsaturated in one position (15, 35, 36). In contrast, Burkholderia kururiensis KP23 produces *Gammaproteobacteria*-like rhamnolipids containing mainly C_10_-C_10_ residues (24).

Rhamnolipid production has not been extensively explored in species of the phyla *Firmicutes*, *Deinococcus-Thermus*, *Actinobacteria*, and *Ascomycota*. Most promising are the results presented for *Thermus* species belonging to the phylum *Deinococcus-Thermus*. Pantazaki et al. (29) produced HAAs and rhamnolipids with chain lengths of 8 to 14 carbon atoms with Thermus thermophilus HB8. Rezanka et al. (30) reported the production of rhamnolipids by *Thermus* sp. strain CCM 2842, mainly containing the C_16_-C_16_ HAA congener, which has not been previously reported. Both groups used selective mass spectrometric methods.

A number of papers in the scientific literature report the synthesis of novel rhamnolipids with novel hosts, which we could not confirm, revealing the need for standardization and guidelines for determination of rhamnolipid and HAA structures. In contrast to rhamnolipids, only a few methods also cover HAAs. Again, HPLC-MS/MS is the method of choice to cover both rhamnolipids and HAAs (37, 38). The most comprehensive HPLC-MS/MS method focusing on HAA was presented by Lépine et al. (39). Therefore, our approach was to apply known and potential *rhlA* genes, express them recombinantly in Escherichia coli, and subject the resulting HAAs to a tailored HPLC-MS/MS analysis for confirmation.

The focus of our study was to explore the diversity of RhlAs and their potential to produce “designer HAAs.” The results are discussed in a phylogenetic context.

## RESULTS

The natural diversity of RhlA, the acyltransferase of the rhamnolipid synthesis pathway, was investigated and exploited for the synthesis of the lipophilic intermediate HAA. We cloned eight *rhlA* homologs drawn from the full phylogenetic range of *Proteobacteria* into the Escherichia coli expression vector pET28a. Alternative RhlAs allowed the synthesis of different HAA congeners.

### Phylogeny of RhlA.

**FIG 3:**
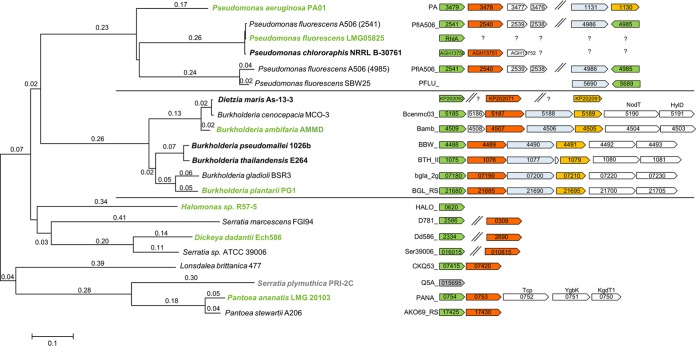
Phylogenetic tree based on the amino acid sequences of RhlA. Operons associated with rhamnolipid formation are drawn next to the organism names, and genes are labeled with their gene locus or protein accession number. Organisms chosen for HAA production in this study are highlighted in green, while elsewhere-confirmed RhlAs are marked in bold. Others were chosen based on homology searches. *S. plymuthica* is marked in gray, as we could not confirm an RhlA activity. Double slashes depict independent genomic locations. In Dietzia maris, the synteny of *rhlABC* is not published. The strains P. fluorescens LMG05825 and P. chlororaphis NRRL-B-30761 are not genome sequenced; therefore, the putative homologous genes are indicated by question marks. The genes for rhamnolipid formation in P. aeruginosa are typically organized in two operons; *rhlB* (red) is located downstream of *rhlA* (green) and encodes rhamnosyltransferase I, which is necessary for mono-rhamnolipid formation. The genes *rhlA* and *rhlB* are colocalized with the regulator and inducer genes (*rhlRI*, white) that are involved in regulation via quorum sensing. In a second operon, *rhlC* (orange), the gene coding for rhamnosyltransferase II, is clustered with a putative transporter (light blue) gene. In the strains P. fluorescens LMG05835 and P. chlororaphis, only the genes shown are sequenced. In the *P. ananatis* LMG20103 operon containing *rhlAB* homologs, three genes are present that code for a methyl-accepting chemotaxis citrate transducer (*tcp*), a putative inner membrane protein (*ygbK*), and a 2-keto-3-deoxygluconate permease (*kgdT1*). In the *Burkholderia* species, the structural genes for di-rhamnolipid formation are organized in a single operon that further includes the genes *nodT* and *hylD*, which are potentially involved in the drug resistance systems of the cell. The tree was constructed using the neighbor-joining method in MEGA7 with default settings. Branch lengths shorter than 0.02 are omitted.

It has been shown that HAA synthesis in E. coli relies only on a recombinantly synthesized RhlA from P. aeruginosa (8, 32). Further, the experimental evidence strongly supports that RhlA selectively determines the β-hydroxy fatty acid chain lengths in HAAs (20). As a first step toward tailor-made HAAs, the natural genetic diversity of RhlA was investigated. Representative RhlA protein sequences for all phyla that were detectable by homology searches in GenBank and KEGG were collected. First, the RhlA of P. aeruginosa was used as a template. As the RhlAs from, for example, *Pantoea* species have limited homology with the protein from P. aeruginosa, homology searches with these sequences were also performed. All identified RhlA proteins are from the classes *Betaproteobacteria* and *Gammaproteobacteria* (Fig. 3). Strains from other phyla that are reported to produce rhamnolipids have not been sequenced, and the genes encoding their rhamnolipid synthesis pathways are not known, with two exceptions; an RhlA (GenBank accession number KP202092) was found in the *Actinobacteria* strain Dietzia maris As-13-3 (28), and the genome sequence of the *Deinococcus-Thermus* strain T. thermophilus HB8 (29) is known. However, in the latter genome, no *rhlA* homolog was found.

In general, the identified RhlAs can be divided into three main branches of a currently sparse phylogenetic tree (Fig. 3). In the first branch, the representatives of the genus *Pseudomonas* form a monophyletic lineage. In the P. aeruginosa strains, represented by strain PA01, two operons containing structural genes for rhamnolipid synthesis are known. In the first of these operons, *rhlA* and *rhlB*, the relevant genes for mono-rhamnolipid synthesis, are clustered with a regulator and inducer for quorum sensing, while *rhlC*, which enables the strain to produce di-rhamnolipids, is located in a different operon and is clustered with a putative transporter (40). Surprisingly, an analysis of the genetic environment of *rhlA* homologs detected using BLAST in the Pseudomonas fluorescens group showed two possible locations. Besides the colocalization with *rhlB*, an *rhlA* homolog is found in synteny with a putative transporter. In P. fluorescens strain A506, *rhlA* genes are present in both loci, while in P. fluorescens strain SBW25, only the latter location and no *rhlB* homolog can be found. Most P. fluorescens strains do not carry the genes for rhamnolipid synthesis (*rhlA* in synteny with *rhlB*, data not shown).

In the second branch, all representatives of the *Burkholderia* genus and the only *Actinobacteria* species, *D. maris* As-13-3 (28), are present. However, the RhlA of *D. maris* As-13-3 is reported to share 96% sequence identity with a Burkholderia cenocepacia protein, indicating that horizontal gene transfer is a probable explanation for its occurrence. In general, in *Burkholderia*, *rhlAB* are located on chromosome II and are in synteny with the putative transporter gene and *rhlC*. Furthermore, *nodT* and *hylD*, coding for enzymes related to efflux and secretion processes, are colocated. In *B. cenocepacia* and Burkholderia ambifaria, an open reading frame encoding a methyl transferase is placed between *rhlA* and *rhlB*. A second operon for rhamnolipid formation exists in Burkholderia pseudomallei and Burkholderia thailandensis on chromosome I (not shown).

The third branch includes homologous proteins from representatives of the orders *Enterobacterales* and *Oceanospirillales*, the latter with the only representative being *Halomonas*. In general, in this branch, the homology of the RhlA proteins is more divergent than in the *Pseudomonas* and *Burkholderia* branches. An *rhlAB*-like operon is found only in *Pantoea* strains (34) and Lonsdalea britanica, while *rhlA* homologs are found in *Serratia* and *Dickeya* strains but not in synteny with an *rhlB* homolog. No experimental evidence for HAA or rhamnolipid formation exists for the organisms in this branch, with the exception of Pantoea ananatis BRT175 (*P. ananatis*) producing the glucolipid ananatoside A, the hydrophobic part of which is an HAA molecule (34, 41). In *P. ananatis* LMG20103, the three genes *tcp*, *ygbK*, and *kgdT1*, which code for a putative methyl-accepting chemotaxis citrate transducer, an effector protein, and a 2-keto-3-deoxygluconate permease, respectively, are encoded in one common operon with the *rhlAB* homologs.

**FIG 4:**
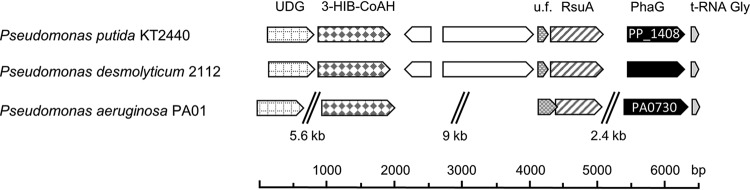
Gene synteny of a *phaG* homolog in *Pseudomonas desmolyticum*. The gene synteny of the *phaG* homolog in *P. desmolyticum* is the same as in P. putida. Homolog genes coding for a uracil-DNA glycosylase (UDG), a 3-hydroxyisobutyryl-CoA hydrolase (3-HIB-CoAH), a protein of unknown function (u.f.), and a ribosomal small subunit pseudouridine A (RsuA) located upstream of *phaG* can also be found in the upstream region of *phaG* in P. aeruginosa. A tRNA homolog is placed downstream. This difference in synteny can be used as a criterion to distinguish *rhlA* from *phaG* in *Pseudomonas* species.

Determining the synteny of sequences identified by BLAST analyses using an RhlA query requires detailed analysis to distinguish RhlA from the transacylase PhaG, an enzyme that links *de novo* fatty acid and polyhydroxyalkanoate (PHA) biosynthesis (42–44) by catalyzing the reesterification from acyl carrier protein (ACP) to CoA. In P. aeruginosa, the protein sequences of RhlA and PhaG have a 44% sequence identity (44), which is similar to the 44 to 48% identity between *Burkholderia* RhlAs and RhlAs of P. aeruginosa. Fig. 3 shows that *rhlA* in *Pseudomonas*, *Burkholderia*, and *Pantoea* is part of a glycolipid synthesis operon. In contrast, *phaG* is located upstream of a tRNA gene, and furthermore, homologs of four of the six upstream genes of *phaG* in Pseudomonas putida can also be found upstream of *phaG* in P. aeruginosa (Fig. 4). We used this difference in the synteny of *rhlA* and *phaG* as a criterion for the identification of rhamnolipid genes in the reported rhamnolipid producer Pseudomonas desmolyticum NCIM-2112. We were especially interested in this strain, as it was reported to produce rhamnolipids with chain lengths of six to eight carbon atoms (45), a congener range not confirmed yet for an isolated RhlA. Full genome sequencing allowed a BLAST search for RhlA; however, only the transacylase-encoding *phaG* was identified. A gene encoding RhlB was not found in the genome of *P. desmolyticum* (data not shown). To improve the authoritative value of rhamnolipid literature, genetic evidence could be, besides high-quality analytics, a means to reduce or ideally avoid miscommunication of rhamnolipid-producing strains (46).

Considering gene synteny, the *rhlA* homologs identified by BLAST analysis of the *Serratia*, *Dickeya*, and *Halomonas* species are not well supported. Experimental evidence should confirm or disprove the RhlA activity.

### HAA synthesis with recombinant E. coli.

**FIG 5:**
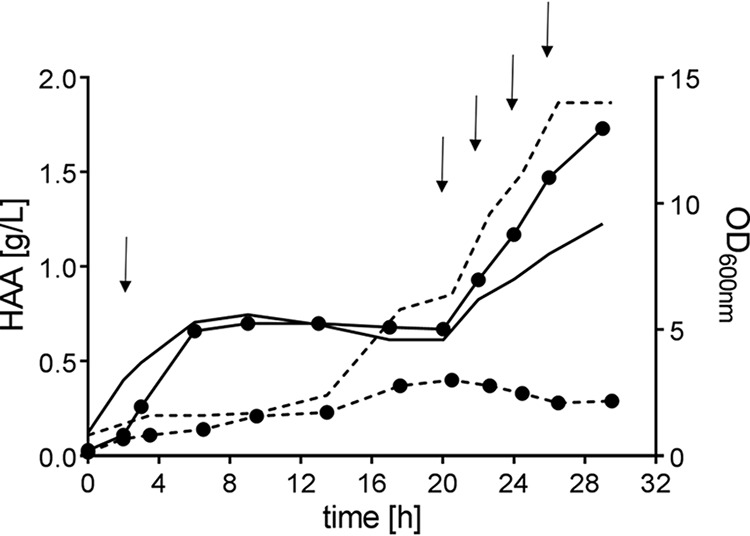
HAA formation (circles) by E. coli BL21(DE3) (dashed lines) and E. coli C43(DE3) (solid lines) transformed with plasmid pPA2 carrying *rhlA* of Pseudomonas aeruginosa PA01. Timepoint 0 indicates induction by IPTG. The OD is depicted without symbols. Glucose (0.2%, wt/vol) was supplemented after sampling to the time points marked by arrows. Each experiment was performed in duplicate.

E. coli strains BL21(DE3) and C43(DE3), each equipped with the *rhlA* gene from P. aeruginosa (pPA2), were grown in LB medium. Defined glucose pulses were given to provide an additional carbon source. When applying E. coli C43(DE3) as the host, glucose addition caused a steep increase in HAA titers 2 h after induction, which subsequently stagnated as glucose was depleted (6 h) (Fig. 5). The high HAA formation and growth rates were restored after the second glucose pulse at 20 h. While the growth rate slowed down 2 h later, the HAA production rate remained high, pointing to the fact that resources were efficiently allocated to the HAA synthesis pathway and diverted from supplying the growth machinery. With this strategy, an HAA titer of 1.7 g/liter 30 h after induction was reached, which is the highest concentration reported so far using recombinant microorganisms for HAA synthesis. Using E. coli BL21(DE3) as the host, the glucose supplementation had no enhancing effect on HAA formation at any time but was used for biomass formation. In this host, only 0.4 g/liter was achieved 20 h after induction.

The main HAA congeners synthesized by E. coli C43(DE3) pPA2 (Table 1) were the same as those produced by a recombinant P. putida KT2440 using the P. aeruginosa *rhlA* (11, 20) or the wild-type P. aeruginosa strain (12, 47–49). The HAA spectrum observed is, however, broader in E. coli, expanding to C_14_-containing congeners. The results support previous data showing that the RhlA enzyme is mainly responsible for HAA congener selectivity, while the host organisms play only a minor role.

**TABLE 1:** Fractions of overall HAA congeners produced in E. coli C43(DE3) detected by HPLC-MS/MS

Identity	*m/z*	Congener^*a*^	Fraction (%) for^*b*^:						
			pPA2	pFLU	pDAD	pANA	pHAL	pAMB	pBUG
14:0	273	C_4_-C_10_*				0.1			
16:0	301	C_6_-C_10_				0.1			
18:0	329	C_8_-C_10_	4.1	0.1	1.9	5.5			
19:0	343	C_9_-C_10_				0.1			
20:0	357	C_8_-C_12_	0.3		0.3	0.3			
20:0	357	C_10_-C_10_	**49.5**	3.0	4.5	**31.3**			
20:1	355	C_8_-C_12:1_			0.1	0.3			
20:1	355	C_10_-C_10:1_	0.3			0.2			
21:0	371	C_10_-C_11_				0.1			
22:0	385	C_8_-C_14_*	0.3				0.3		0.2
22:0	399	C_10_-C_12_	**32.5**	**17.1**	7.1	**16.4**	0.1	0.3	0.2
22:1	383	C_8_-C_14:1_*		0.4			0.1		0.1
22:1	383	C_10_-C_12:1_	4.2	**21.3**	5.6	**10.7**			
23:0	399	C_10_-C_13_*		0.3	0.3	0.2		0.1	
24:0	413	C_10_-C_14_	4.1	**23.8**	**25.8**	**10.0**	2.0	2.3	4.5
24:0	413	C_12_-C_12_	0.4	1.0		1.5	0.5	0.2	0.1
24:1	411	C_10_-C_14:1_	2.9	**25.2**	**47.4**	**10.8**	2.0	0.6	3.0
24:1	411	C_12_-C_12:1_				1.5			
24:2	409	C_10:1_-C_14:1_			0.2				
25:0	427	C_11_-C_14_*						0.3	0.1
25:0	427	C_12_-C_13_*					0.1	0.6	
25:1	425	C_10_-C_15:1_*			0.1				
25:1	425	C_11_-C_14:1_*						0.1	
26:0	441	C_10_-C_16_		0.2		0.1			
26:0	441	C_12_-C_14_		1.2	1.2	1.5	**10.1**	**12.1**	5.1
26:0	441	C_13_-C_13_*					0.2	1.2	
26:1	439	C_10_-C_16:1_		4.1	2.6	3.3	0.1	0.1	0.4
26:1	439	C_12_-C_14:1_		1.2	1.6	2.2	8.8	4.9	3.9
26:2	437	C_12:1_-C_14:1_		0.3	0.6	0.7	0.1		0.1
27:0	455	C_12_-C_15_*						0.1	
27:0	455	C_13_-C_14_					0.4	6.3	0.6
27:1	453	C_13_-C_14:1_*					0.3	4.0	0.4
28:0	469	C_14_-C_14_				0.1	**22.1**	**12.2**	**24.4**
28:1	467	C_10_-C_18:1_*		0.4		0.7			
28:1	467	C_12_-C_16:1_*		0.2	0.1	0.3	0.3		0.3
28:1	467	C_14_-C_14:1_			0.1	0.4	**30.8**	**36.5**	**28.3**
28:2	465	C_12:1_-C_16:1_*				0.2			
28:2	465	C_14:1_-C_14:1_			0.2	0.2	**14.9**	**10.3**	**11.7**
29:0	483	C_13_-C_16_*					0.1		
29:0	483	C_14_-C_15_							0.3
29:1	481	C_13_-C_16:1_*						0.2	
29:1	481	C_14_-C_15:1_*					0.1	0.5	0.2
29:1	481	C_14:1_-C_15_*						0.5	
29:2	479	C_14:1_-C_15:1_						0.4	
30:0	497	C_14_-C_16_					0.5		1.2
30:1	495	C_12_-C_18:1_*				0.1			
30:1	495	C_14_-C_16:1_				0.2	3.5	1.7	8.9
30:2	493	C_12:1_-C_18:1_*				0.1			
30:2	493	C_14:1_-C_16:1_*				0.2	2.1	4.2	4.4
32:1	523	C_14_-C_18:1_							0.5
32:1	523	C_16_-C_16:1_					0.1		0.1
32:2	521	C_14:1_-C_18:1_*				0.1	0.1		0.4
32:2	521	C_16:1_-C_16:1_					0.1		0.2
Average chain length			10.5	11.6	11.8	10.8	13.8	13.7	14.0

a New congeners are marked by an asterisk. Congeners include both variants for chain positions, e.g., C_8_-C_10_ and C_10_-C_8_.

b The main congeners for each plasmid are in bold font. pPA2, P. aeruginosa PAO1; pFLU, P. fluorescens LMG 05825; pDAD, *D. dadantii* Dd586_2334; pANA, *P. ananatis* LMG 20103; pHAL, *Halomonas* sp. R57-5; pHAL, *Halomonas* sp. R57-5; pAMB, *B. ambifaria* AMMD; pBUG, *B. plantarii* (*glumae*) PG1.

### Diversification of the HAA spectrum by exploiting natural genetic variance.

In order to increase HAA congener diversity, seven additional *rhlA*s of species representing the identified evolutionary space were used.

The first obvious choice from the *Betaproteobacteria* was RhlA of Burkholderia plantarii PG1 (formerly Burkholderia glumae PG1), which synthesizes mainly C_14_ rhamnolipids (20, 21). We also chose RhlA of *B. ambifaria*, which was of particular interest, as the protein shares 91% identity with RhlA of *D. maris*, which was reported to produce the C_10_-C_10_ congener (28). Our purpose was to verify this nontypical main congener for the *Burkholderia* genus with pAMB.

In contrast to the 16S rRNA phylogeny, in which *Enterobacterales*, *Oceanospirillales*, and *Pseudomonas* are classes of *Gammaproteobacteria*, the RhlA sequences of the *Enterobacterales* and the *Oceanospirillales*-representative *Halomonas* form a common third branch (Fig. 3). We thus selected RhlA from *P. ananatis* LMG20103 as the first representative from the *Enterobacterales* branch. This strain is fully genome sequenced, and the genes for glycolipid synthesis are present (34, 50). Rooney et al. (17) and Hošková et al. (18) reported that other *Enterobacterales* synthesize rhamnolipids with mainly C_10_-C_10_ HAAs (Fig. 2). The N terminus of RhlA in *P. ananatis* is longer than those of other RhlA proteins, which might be due to automated annotation. For this reason, two versions of *rhlA* were cloned, one representing a normal-sized *rhlA* and the long *rhlA* version. Both *rhlA*s led to HAA production (data for the long version not shown), suggesting that the normal-sized RhlA is the native protein. Additionally, sequencing indicated a frameshift in the published sequence that led to 13 incorrectly annotated amino acids. A comparison with RhlA from, e.g., Pantoea stewartii A206 confirms this finding, and a corrected sequence was submitted to GenBank (accession number MF671909). As mentioned above, the gene synteny in Dickeya dadantii Ech586, *Halomonas* sp. R57-5, and Serratia plymuthica PRI-2C does not show colocalization with genes related to glycolipid synthesis, but the *rhlA* homologs are isolated in the genome. To experimentally confirm the activity, we further investigated HAA formation using *rhlA* genes of these strains.

Finally, we included the *rhlA* from P. fluorescens LMG 05825 (P. chlororaphis ATCC 17813), which is reported to be the same strain as P. chlororaphis NRRL B-30761 (35), a strain producing mainly C_10_-C_12_ and C_10_-C_12:1_ congeners (15). Solaiman et al. (36) found an operon containing *rhlAB* and the regulator gene *rhlR* (Fig. 3) in this strain. While we could confirm the previous results, the *rhlA* from strain LMG05825 carried two nucleotide changes resulting in one amino acid difference in RhlA.

E. coli strains were equipped with one of the eight *rhlA* genes and cultivated as described above. Glucose was fed 2 and 22 h after IPTG (isopropyl-β-d-thiogalactopyranoside) induction. Seven of the eight recombinant strains produced HAAs (Table 1); E. coli C43(DE3) pPLY was the exception. Again, while the main HAA congeners were highly similar to reported congener compositions of wild-type strains, the congener spectrum might be a bit wider, which however, could also be a result of the sensitive method used for identification in this study. By the combination of efficient chromatographic separation and structure informative tandem mass spectrometric detection, the resulting HPLC-MS/MS method enables selective and sensitive detection of HAAs. A limit of detection in the range of 0.1 mg/liter is achieved, and thus, HAA with a relative share of <0.1% can be detected (Table 1).

As expected from mono-rhamnolipids produced by the wild-type strain P. chlororaphis NRRL-B-30761 (15), our congener determination with plasmid pFLU revealed a different main congener spectrum than with pPA2 from P. aeruginosa. Accordingly, we detected C_10_-C_12_ and C_10_-C_12:1_ to be among the main congeners, but additionally, we identified C_10_-C_14_ and C_10_-C_14:1_ to be present in even slightly larger fractions. The C_10_-C_14_ congener was also detected by Gunther et al. (15), though in a smaller fraction. In contrast to pPA2, where the longest detected chain contained 14 carbon atoms, with pFLU, congeners containing C_16_, C_16:1_, or even C_18:1_ chains were present.

**FIG 6:**
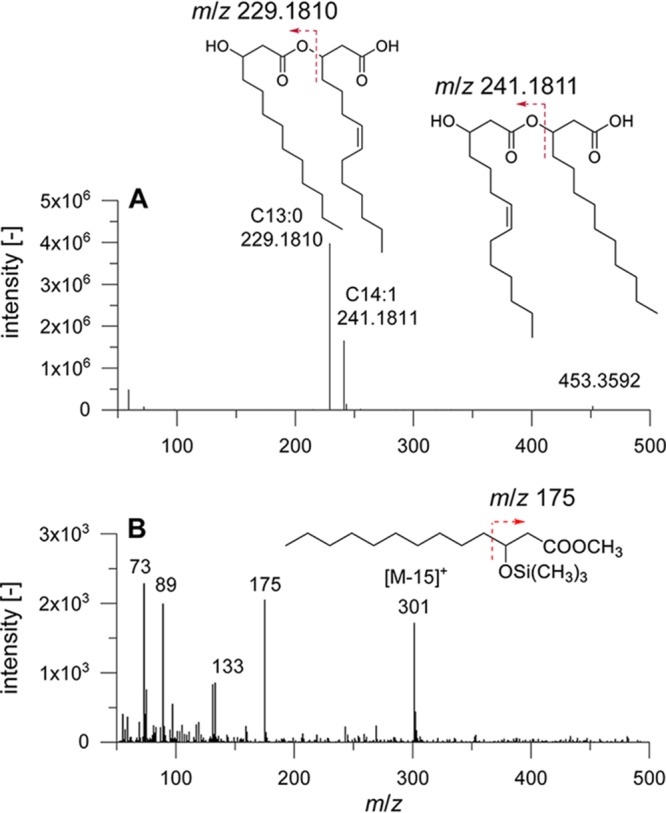
(A) HPLC high-resolution MS/MS spectrum of HAA 27:1. The fragments correspond to C_13:0_ and C_14:1_ hydroxy fatty acids as indicated by the shown structures. Please note that the double-bond position in C_14:1_ could not be determined using this method. (B) GC-MS spectrum of C_13:0_ hydroxy fatty acid after hydrolysis, methylation, and trimethylsilylation to confirm the presence of odd-chain hydroxy fatty acids. The chain length is determined by the characteristic [M-15]^+^ ion, while the diagnostic fragment (*m/z* 175) indicates the position of the hydroxy group.

The two RhlA proteins from the *Betaproteobacteria* branch (pBUG and pAMB) showed 14 carbon atoms in both chains. For pBUG, this was expected due to the phylogenetic classification with other *Burkholderia* strains, for which C_14_-C_14_ rhamnolipid production has been reported in wild-type (19, 21–23) and recombinant strains (20). With pBUG, 16% of the HAAs incorporated at least one C_16_ or C_16:1_ fatty acid. In the phylogenetic tree shown in Fig. 3, pAMB is arranged with RhlA from Dietzia maris (28), shown to produce C_10_-C_10_-containing rhamnolipids. Therefore, the result with mainly C_14_ chain lengths was unexpected. Besides the main fraction of C_14_ chain lengths, we found with this plasmid the most significant fraction of unusual congeners containing chain lengths with odd numbers, namely, 12% containing at least one chain with 13 carbon atoms and in traces C_15_ or C_15:1_. To further confirm the presence of these odd-numbered hydroxy fatty acids, we conducted LC-MS/MS measurements applying high-resolution MS. Besides high resolution, the instrument used also delivers high mass accuracy (<5 ppm relative mass deviation compared to the theoretical value). Hence, elemental compositions can be deduced not only from the intact HAA molecule but also for the fragments in MS/MS mode. Exemplar data are presented in Fig. 6A, where the high-resolution MS/MS mass spectrum of an HAA molecule containing 27 carbon atoms with 1 unsaturation (HAA 27:1) after HPLC separation is depicted. Collision-induced dissociation of the precursor ion (measured *m/z* 453.3592, calculated *m/z* 453.3586, 1.3 ppm) gave rise to two complementary fragments (measured *m/z* 229.1810, calculated *m/z* 229.1809, 0.4 ppm and measured *m/z* 241.1811, calculated *m/z* 241.1809, 0.8 ppm). These fragments confirm the presence of C_13:0_ and C_14:1_ hydroxy fatty acids. The fragmentation is assigned according to Lépine et al. (39). Therefore, the detection of these two fragments also demonstrates that two congeners are contained, i.e., HAA C_13:0_-C_14:1_ and HAA C_14:1_-C_13:0_. Furthermore, the presence of odd-chain hydroxy fatty acids was confirmed using complementary gas chromatography-mass spectrometry (GC-MS) analysis. HAA samples were hydrolyzed and derivatized to yield the corresponding methyl ester. Additional trimethylsilylation of the hydroxy group facilitated the assignment of chain length as well as position of the hydroxy group in the mass spectrum obtained by electron ionization (Fig. 6B).

Most surprising and divergent were the results we obtained with the plasmids from the *Enterobacterales* and *Halomonas* species. Though the RhlA homologs form their own branch (Fig. 3), the HAAs detected with the single plasmids do not show common characteristics. Again, for pPLY, HPLC-MS/MS did not confirm HAAs in the culture supernatant but did confirm other fatty acids. Notably, these free fatty acids experienced similar retention on the used HPLC column as HAAs. Using unspecific detection, such as charged aerosol detection, or by evaporative light scattering detection, false annotation cannot be ruled out. With plasmid pDAD, a comparable spectrum to pFLU was observed. Strikingly, the main fraction contained C_10_-C_14_ or C_10_-C_14:1_ (26 and 47%, respectively), indicating a high specificity for these congeners. With pANA, the main congeners contained C_10_-C_10_ (31%), C_10_-C_12_ or C_10_:C_12:1_ (27%), and C_10_-C_14_ or C_10_-C_14:1_ (21%), which is comparable to the congeners found with pPA2 and pFLU. Plasmid pHAL, in contrast, showed congeners like the *Burkholderia* strains with saturated and monounsaturated C_14_ chains.

### Novel HAA congeners.

In total, we detected 53 congeners, 23 of which have not been reported before (see marked congeners in Table 1). In general, the new congeners can be categorized into three characteristics or combinations thereof. In the first, HAA molecules consist of two chains differing in four or more carbon atoms in length; in the second, chains with so far unknown monounsaturations can be found; and in the third, odd chain lengths occur. For the first category, we found C_4_-C_10_, C_8_-C_14_, C_8_-C_14:1_, C_10_-C_15:1_, C_10_-C_18:1_, and C_12_-C_16:1_. The chain lengths of the congeners C_12_-C_18:1_, C_12:1_-C_18:1_, and C_14:1_-C_18:1_ have been reported in rhamnolipids produced by *Thermus* sp. CCM 2842 (30), where the molecules were saturated. Other new congeners in the second category with monounsaturations are C_8_-C_14:1_, C_12_-C_16:1_, C_12:1_-C_16:1_, and combined with the third category including odd chain lengths, C_10_-C_15:1_, C_11_-C_14:1_, C_13_-C_14:1_, C_13_-C_16:1_, C_14_-C_15:1_, C_14:1_-C_15_, and C_14:1_-C_15:1_. More new odd chain lengths are C_11_-C_14_, C_11_-C_14:1_, C_12_-C_13_, C_10_-C_15:1_, C_13_-C_13_, C_12_-C_15_, C_13_-C_14:1_, and C_13_-C_16_. For the third category, it has to be mentioned that other odd chain lengths were detected earlier (30, 37, 51). In our results, it is striking that 7.7% of the overall congeners are new congeners with odd numbers synthesized by E. coli C43(DE3) pAMB.

The congeners that were produced covered the entire HAA spectrum known in wild-type *Proteobacteria* species. The congener C_8_-C_8_ produced by P. aeruginosa 57RP (49, 52) was found in some, but not all, experiments with pPA2 and hence is not listed in Table 1.

## DISCUSSION

The esterification of (hydroxy-) fatty acids as it is catalyzed by RhlA is a rare enzyme activity. A similar activity can be found in the black yeast fungus Aureobasidium pullulans. In this strain, liamocin, a glycolipid consisting of mannitol linked with three or four 3,5-dihydroxydecanoic ester groups, is produced (53). Our survey for RhlA in microorganisms showed its presence mainly in the *Betaproteobacteria* and *Gammaproteobacteria* phyla, with little evidence in other phyla. We exploited the natural diversity of RhlA, allowing the synthesis of distinct HAA congener mixtures using E. coli as a host. The confirmed substrate specificity of RhlA opens the door for the production of tailor-made HAAs.

Fig. 2 shows that rhamnolipid producers are not restricted to representatives of the *Betaproteobacteria* and *Gammaproteobacteria* phyla. However, we and others (46, 54) have experienced difficulties in reproducing and confirming previous studies showing rhamnolipid synthesis by bacteria of different phyla. In many cases, we did not detect rhamnolipid production and/or genetic evidence for rhamnolipid synthesis despite having cultivated and/or sequenced the reported rhamnolipid producers, respectively. Having had similar experiences, Irorere et al. (46) ascertained that unequivocal analytical techniques to determine rhamnolipid production were not used and concluded that particular reports might be erroneous. As mentioned above, Jadhav et al. (45), for example, reported that *P. desmolyticum* NCIM-2112 produced mono-rhamnolipids with fatty acid chain lengths of from six to eight carbon atoms. Our efforts to identify an *rhlA* homolog after genome sequencing failed. We cultivated the organism as described by the authors but detected no rhamnolipids using HPLC-MS/MS (data not shown). The question of whether *P. desmolyticum* encodes an enzyme with RhlA activity but of a different phylogeny remains, which is consistent with the observations of Kügler et al. (54) finding no evidence of reports of rhamnolipid production by *Actinobacteria*. Indeed, we found no RhlA with homology searches in non-*Proteobacteria* species, with the exception of the actinobacterium Dietzia maris AS-13-3. A detailed survey of the analytical methods applied for the identification of novel rhamnolipid-producing strains is necessary. In this regard, the reports of rhamnolipid production in Renibacterium salmoninarum 27BN (27), Tetragenococcus koreensis JS (25), and *Aspergillus* sp. strain MFS1 (31) do not fulfill the criteria for unequivocal rhamnolipid identification proposed by Irorere et al. (46).

### The diversity of HAA congeners might be broadened by identifying RhlAs from *Betaproteobacteria* and *Gammaproteobacteria*.

The evolutionary relationships between the known RhlAs (Fig. 3) are to a large extent consistent with the species phylogeny based on 16S rRNA gene sequences (Fig. 2). However, it is striking that the RhlA proteins within the genus *Pseudomonas* do not form a monophyletic lineage with the RhlAs from the *Enterobacterales* (*Serratia*, *Dickeya*, *Lonsdalea*, and *Pantoea*) as the 16S rRNA genes do. While pseudomonads and *Enterobacterales* are both *Gammaproteobacteria*, the RhlA proteins of the *Enterobacterales* are outgrouped, forming a separate branch.

Within the pseudomonads, species-specific HAA congeners could be produced. While pPA2 is the most prominent C_10_-C_10_ producer, we detected with pFLU a C_12_ or C_14_ chain combined with a C_10_ chain, which confirms the findings of Gunther et al. (15). However, Gunther found C_10_-C_12_ or C_10_-C_12:1_ as the main congener; in our study, C_10_-C_14_ and C_10_-C_14:1_ turned out to be even more prominent.

The *Burkholderia* species *B. plantarii*, B. thailandensis, and *B. mallei* synthesize mainly C_14_-C_14_ rhamnolipids (19, 21–23, 55). This was confirmed in this study. Using the *B. plantarii* RhlA, the average carbon chain was determined to have 14.0 carbon atoms. However, in terms of RhlA diversity, the phylogeny of the known RhlA proteins indicates that other *Burkholderiaceae* might produce HAAs with shorter fatty acids. *B. kururiensis*, belonging to the genus *Paraburkholderia* (24), is reported to mainly produce the C_10_-C_10_ rhamnolipid congeners. Two explanations for this finding are possible. On the one hand, a C_10_-specific protein might have been transferred to *B. kururiensis* from, e.g., P. aeruginosa via horizontal gene transfer. On the other hand, the *rhlA* might have evolved from the original *Burkholderia* type *rhlA* to be more promiscuous toward shorter fatty acid chain lengths.

Most new congeners with odd chain lengths were produced when RhlA from *B. ambifaria* was applied, which we confirmed with GC analytics (Fig. 6B). It was shown that in contrast to acetyl-CoA, propionyl-CoA can be accepted by the enzyme FabH as a precursor to chain elongation, resulting in odd-chain-length fatty acids (56, 57). FabH varies depending on its bacterial origin and accepts acetyl-CoA or propionyl-CoA in bacteria synthesizing straight-chain fatty acids, while in branched-chain fatty acid-producing bacteria, branched-chain acyl-CoAs serve as precursors for chain elongation (57). Our results showing straight C_13_ chains when using pAMB in E. coli indicate that FabH of E. coli is of the straight-chain type delivering the substrate for the *B. ambifaria* RhlA.

Most interesting and representing the group with the most potential toward novel HAA congeners are the results we obtained with RhlA proteins from the *Enterobacterales* and *Halomonas*. Except for *Pantoea* and *Lonsdalea*, the genes coding for RhlAs in this group are not colocalized with other genes related to glycolipid formation and thus are difficult to distinguish from *phaG*. In contrast to the *Pseudomonas* and *Burkholderia* branches shown in Fig. 3, RhlA homologs from five genera are combined in the third branch. With only four RhlAs tested, we found a diversity within this group ranging from no HAAs with pPLY over similar congeners like in the pseudomonads (pDAD and pANA) to a *Burkholderia*-like spectrum with pHAL. So far, few results from *Enterobacterales* have been presented in the literature. Reports about rhamnolipid formation by the wild-type strains Enterobacter asburiae (17, 18), Enterobacter hormaechei, P. stewartii (17) (Fig. 1), and *P. ananatis* BRT175, a strain producing a glycolipid with a sugar moiety other than rhamnose (34, 41), show similar fatty acid chain lengths to those we detected with pANA and pDAD. Though the HAA spectrum from, e.g., pDAD and pFLU or pHAL and pBUG are similar, the RhlAs are only distantly related and not arranged in the same phylogenetic lineage. The diversity of HAAs within the *Enterobacterales* indicated by long branch lengths hints at the existence of more proteins with RhlA activity in this and other orders of the *Gammaproteobacteria*, such as the *Oceanospirillales*. With confirmed RhlA activity from numerous species, the sparse tree depicted in Fig. 3 might develop toward distinct branches related to genera. A tendency can already be seen with our data obtained with pHAL, pDAD, pPLY, and pANA. Eliminating the unconfirmed *S. plymuthica* strain, to date, three strains from *Lonsdalea* and *Pantoea* form their own lineage. In these species, a colocalization of the *rhlA* homologs with an *rhlB* homolog are found.

Our results indicate that the *rhlA* genes are conserved within microbial genera. Because RhlA mainly determines substrate specificity in the rhamnolipid synthesis pathway, the main fatty acid congener of HAAs and rhamnolipids can be inferred from knowledge of the species of the producing organism. Insights into the correlation between microbial and RhlA phylogeny and RhlA specificity may be fostered by additional genomic and production data from rhamnolipid producers, ideally increasing the number of HAA-producing species and genera for which genetic evidence for *rhlA* genes exists.

### HAAs synthesized by non-*Proteobacteria*.

Toribio et al. (58) argued in 2010, when hundreds of genomes were already available in databases, that the rare occurrence of *rhlA* homologs outside of the *Betaproteobacteria* and *Gammaproteobacteria* species suggested that horizontal gene transfer occurs only in rare circumstances. This conclusion agrees with the results for *D. maris* presented here (28). Although 360,000 genomes are currently available in the Genomes On Line Database (GOLD), and many more are available in others, the early observation by Toribio et al. (58) is still valid. Although no evidence is presented here, a massive gene loss in most other phyla and genera cannot be excluded. With BLAST searches, RhlAs cannot be detected in, e.g., *Thermus*. Despite having a common ancestor, it is possible that the phylogenetic distance increased during evolution. This hypothesis is supported by the fact that the *Betaproteobacteria* RhlA proteins from P. aeruginosa and *P. ananatis*, which show a similar HAA spectrum, share a mere 35% identity or, to name another example, proteins from *B. plantarii* PG1 and *Halomonas* spp. show only 50% identical positions (data not shown). Alternatively, rhamnolipids might be synthesized by proteins that do not share an evolutionary origin with RhlA.

Some evidence exists for alternative genes, especially for strains of the genus *Thermus*, which is encouraging. Pantazaki et al. (29) reported the production of HAAs and rhamnolipids with chain lengths of 8 to 14 carbon atoms using the fully sequenced strain T. thermophilus HB8. No homologs of *rhlA* and *rhlB* were found in the genome using conventional BLAST approaches. The main congener detected in *Thermus* sp. CCM 2842 was Rha-C_16_-C_16_, and fatty acid chain lengths of up to 24 occur in small fractions (30), indicating that an RhlA with different substrate specificity exists; again, no genetic evidence is available. The numerous reports of HAA and rhamnolipid synthesis by species not belonging to *Betaproteobacteria* and *Gammaproteobacteria* remain something of a mystery, with explanations as divergent as erroneous analytics, horizontal gene transfer, massive gene loss, and parallel evolution. The challenge to identifying the genetic origin of rhamnolipid synthesis in phyla such as *Firmicutes*, *Actinobacteria*, and *Deinococcus-Thermus* thus remains.

## MATERIALS AND METHODS

### Bacterial strains, plasmids, and synthetic genes.

*P. ananatis* LMG 20103, P. fluorescens LMG 05825, and *B. ambifaria* LMG 19182 (= *B. ambifaria* AMMD) were purchased from the BCCM/LMG Bacteria Collection, and *P. desmolyticum* NCIM-2112 (45) was purchased from the National Collection of Industrial Microorganisms (NCIM). Plasmid pVLT33_rhlAB_BG_, containing *rhlA* from *B. plantarii* PG1, was kindly provided by A. Wittgens (20). The native gene from *Dickeya* and codon-optimized genes for the *rhlA* homologs from *Halomonas* and *Serratia* were synthesized by IDT (Coralville, IA, USA).

### Construction of expression plasmids.

Eight expression plasmids carrying *rhlA* from either P. aeruginosa PA01, *P. ananatis* LMG 20103, *B. plantarii* PG1, P. fluorescens LMG 05825, *B. ambifaria* LMG 19182, *Halomonas* sp. R57-5, *D. dadantii* Ech586, or *S. plymuthica* PRI-2C were constructed. The *rhlA* gene from P. aeruginosa PA01 was amplified from plasmid pPS05 (59) and cloned into pET28 as described previously (9). The *rhlA* genes of *P*. ananatis LMG20103, P. fluorescens LMG 05825, and *B. ambifaria* LMG 19182, were amplified from genomic DNA and cloned into pET28a, and the *B. plantarii* PG1 *rhlA* was amplified from plasmid pVLT33_rhlAB_BG_. For the construction of plasmids pPLY, pHAL, and pDAD, *rhlA* was amplified from G-strings. Purified PCR products and digested pET28a were used for subsequent Gibson assembly using the respective cloning kits (NEB, Ipswich, MA, USA) following the supplier’s instructions. The plasmids used are listed in Table 2.

**TABLE 2:** Strains and plasmids used in this study

Strain or plasmid	Characteristics/purpose	Reference
Strains		
P. aeruginosa PA01	Wild-type, cloning of *rhlA*	13
P. fluorescens LMG 05825	Wild-type, cloning of *rhlA*, strain used instead of P. chlororaphis NRRL B-30761	15
*P. desmolyticum* NCIM-2112	Wild-type, identification of *phaG*	45
*P. ananatis* LMG 20103	Wild-type, cloning of *rhlA*	34, 41, 50
*B. ambifaria LMG* 19182	Wild-type, cloning of *rhlA*, strain used instead of *P. ambifaria* AMMD	64
E. coli DH5α	Cloning procedures	65
E. coli BL21(DE3)	Expression host	66
E. coli C43(DE3)	Expression host	67
Plasmids		
pPS05	Plasmid harboring *rhlA* from Pseudomonas aeruginosa PA01, SynPro16, Tet^R^	59
pVLT33_rhlAB_BG_	Plasmid harboring *rhlA* from Burkholderia plantarii *(glumae)* PG1, *lacI*, P_tac_ Km^R^	20
pET28a	Expression vector, T7 promoter, His-Tag, *lacI*, pBR322 ori, f1 ori, Km^R^	68
pPA2	*rhlA* from P. aeruginosa PA01 cloned into pET28a (NdeI/SalI), Km^R^	9
pANA	*rhlA* from *P. ananatis* LMG 20103 cloned into pET28a (NdeI/BamHI), Km^R^	This work
pBUG	*rhlA* from *B. plantarii* PG1 cloned into pET28a (NdeI/BamHI), Km^R^	This work
pFLU	*rhlA* from P. fluorescens 05825 cloned into pET28a (NdeI/SalI), Km^R^	This work
pAMB	*rhlA* from *B. ambifaria* 19182 cloned into pET28a (NdeI/BamHI), Km^R^	This work
pHAL	Codon optimized *rhlA* from *Halomonas* sp. R57-5 cloned into pET28a (NdeI/BamHI), Km^R^	This work
pDAD	*rhlA* from *D. dadantii* Ech586 cloned into pET28a (NdeI/BamHI), Km^R^	This work
pPLY	Codon optimized *rhlA* from *S. plymuthica* cloned into pET28a (NdeI/BamHI), Km^R^	This work

### Culture conditions for HAA production.

Plasmids pPA2, pANA, pBUG, pFLU, pAMB, pDAD, pPLY, and pHAL were separately transformed in E. coli. For HAA production, a 100-ml shake flask with 10 ml of LB containing 50 μg/ml kanamycin was inoculated with a freshly transformed E. coli strain. The cells were grown overnight at 37°C in a Multitron shaker (Infors HT, Bottmingen, Switzerland) at 200 rpm with a throw of 25 mm and humidity of 80%. HAA synthesis was conducted in 50 ml of the same medium in a 500-ml shake flask without baffles. The culture was inoculated to an optical density at 600 nm (OD_600_) of 0.1, and cells were grown at 37°C until the OD_600_ was between 0.5 and 0.9. Expression of *rhlA* was induced by the addition of 0.5 mM IPTG, at which point the temperature was lowered to 30°C and the shaking speed raised to 300 rpm to ensure that sufficient oxygen was supplied. Next, 2 and 20 h after the cultures were induced, 200 μl of 50 g/liter glucose was added, and the supernatant for HPLC-MS/MS was harvested 28 h after induction. In the experiment shown in Fig. 5, the cultures received 0.2% (wt/vol) glucose 2, 20, 22, 24, and 26 h after induction.

### Quantification of HAAs.

For HAA quantification, components of the samples were separated using reverse-phase chromatography on a C_18_ column and detected with a charged aerosol detector (RP-HPLC-CAD) as described by Tiso et al. (60), based on the method established by Behrens et al. (61). Harvesting of cells and cell residue was accomplished by centrifugation at 13,000 × *g* for 3 min. Next, 400 μl of the supernatant was mixed with 400 μl of acetonitrile, and the resulting mixture was vortexed. Samples were subsequently incubated overnight at 4°C to facilitate precipitation of any residual material that might clog the column. Prior to HPLC analysis, the samples were centrifuged again, and the supernatant was filtered with a Phenex-RC (regenerated cellulose) syringe filter (diameter, 4 mm; pore size, 0.2 μm; Phenomenex, Torrance, CA, USA).

An UltiMate3000 series HPLC system was used. The instrument is composed of an LPG-3400SD pump, a WPS-3000 (RS) autosampler, a TCC-3000 (RS) column oven, and a Corona charged aerosol detector (CAD) (all Thermo Fisher Scientific, Inc., Waltham, MA, USA). The Corona CAD was supplied with a continuous nitrogen stream by a Parker Balston NitroVap-1LV nitrogen generator (Parker Hannifin GmbH, Kaarst, Germany).

A Nucleodur C_18_ gravity column from Macherey-Nagel GmbH and Co. KG (Düren, Germany) with a precolumn cartridge of 4 mm length, a particle size of 3 μm, and dimensions of 150 × 4.6 mm was utilized to separate the HAAs. The gradient program started with 70% acetonitrile and 30% purified water containing 0.2% formic acid. After this, composition was kept constant for 1 min, and the acetonitrile percentage began to increase and continued until it reached 100% at 8 min. After 11 min of total running time, the acetonitrile percentage was decreased to 70% within 1 min. The total analysis time was 15 min. The column oven temperature was set to 40°C, and 5 μl of the sample was injected. The flow rate was set to 1 ml/min.

### HAA composition analysis by HPLC-MS/MS.

For HAAs produced by E. coli C43(DE3) pPA2, BL21(DE3) pPA2, C43(DE3) pANA, C43(DE3) pFLU, and C43(DE3) pBUG, sample preparation and chromatographic separation were carried out as described by Behrens et al. (37). In short, 2 ml of the sample material [4 ml for the less concentrated E. coli C43(DE3) pBUG sample] was subjected to solid-phase extraction on a strong quaternary ammonium-modified polymeric anion-exchange material (Chromabond HR-XA, 3 ml volume, 200 mg adsorbent weight; Macherey-Nagel GmbH and Co. KG, Düren, Germany). Then, 10 μl of solid-phase extraction (SPE) extract [20 μl for the E. coli C43(DE3) pBUG sample] was analyzed with HPLC using a Nucleodur Sphinx RP column (150 × 2 mm, 3 μm; Macherey-Nagel, Düren, Germany). A mobile phase gradient consisting of 5 mmol/liter aqueous ammonium formate buffer (pH 3.3) containing 5% acetonitrile (vol/vol) (A) and acetonitrile (B) was used. Mass spectrometric detection was carried out with electrospray ionization in negative ionization mode. Structural information was provided by performing additional MS/MS experiments on two different mass spectrometers as follows. Samples of E. coli C43(DE3) pPA2 and C43(DE3) pBUG were analyzed on a Micromass Quattro micro triple quadrupole mass spectrometer (product ion scans) as detailed previously (37). MS/MS characterization of extracts from E. coli C43(DE3) pANA, C43(DE3) pFLU, and BL21(DE3) pPA2 was carried out on a linear ion trap mass spectrometer (LTQ XL; Thermo Fisher Scientific, Inc., San Jose, CA, USA) under the conditions described by Behrens et al. (38).

Additional confirmatory experiments were conducted using high-resolution MS. The analytes were identified by their accurate masses detected on a QExactive hybrid quadrupole Orbitrap (Thermo Fisher Scientific, Waltham, MA, USA) mass spectrometer. The instrument was operated in negative electrospray ionization mode with the following parameters: spray voltage, 3.0 kV; sheath gas, 40 arbitrary units (AU); auxiliary gas, 10 AU; sweep gas, 1 AU; resolution, 140,000× (full width at half maximum [FWHM] at *m/z* 200); and mass range, *m/z* 200 to 1,000.

The intact HAAs were detected as deprotonated molecules ([M-H]^–^); e.g., a peak at *m/z* 301 was observed for C_8_-C_8_ HAA. MS/MS product ion spectra were dominated by the cleavage of the ester bond between the two β-hydroxy fatty acids as described by Lépine et al. (39). The product ion spectrum of the parent ion at *m/z* 301 showed a major fragment at *m/z* 159, which corresponds to a C_8_ fatty acid moiety, thus confirming the assignment of the parent as C_8_-C_8_ HAA. Fragments with *m/z* 131 and 187 were also present. These ions indicate the presence of C_6_ and C_10_ fatty acid moieties, therefore confirming by LC-MS/MS that not only C_8_-C_8_ HAA but also C_6_-C_10_ and C_10_-C_6_ HAAs were present.

### Confirmation of hydroxy fatty acids by GC-MS.

The HAAs were analyzed using gas chromatography-mass spectrometry (GC-MS). Therefore, an aliquot of each sample was dried under a gentle stream of nitrogen and hydrolyzed with 0.5 M NaOH in MeOH-H_2_O solution (9:1 [vol/vol], 2 ml, 70°C, 1 h). Afterward, the solution was acidified to pH 3 with 1 M HCl, and the fatty acids were extracted with chloroform (3 × 3 ml). After removal of the solvent, fatty acid methyl esters (FAMEs) were prepared by adding 100 μl of BF_3_-MeOH (14%, wt/vol) and heating (75°C, 1 h). Then, 2 ml of H_2_O was added, and the FAMEs were extracted with chloroform (3 × 2 ml). The solvent was evaporated using a gentle stream of nitrogen. The residue was redissolved in 25 μl of pyridine and 50 μl of the silylating agent (BSTFA:TMCS [99:1, vol/vol]) and then heated (70°C, 1 h). Finally, the silylating agent was removed under a gentle stream of nitrogen, and the residue was rediluted in 0.2 ml n-hexane and used for GC-MS analysis.

After derivatization, samples were analyzed using a GCMS-QP-2020 equipped with a Nexis GC-2030 gas chromatograph (both Shimadzu, Kyoto, Japan). A 30-m, 0.25-mm-inside-diameter (i.d.), 0.25-μm-film-thickness DB-5MS column (J&W Scientific, Folsom, CA, USA) was used for the separation. Samples (1 μl) were injected using an AOC-20i Plus autosampler (Shimadzu, Kyoto, Japan) and a programmed temperature vaporization (PTV) inlet (250°C) in splitless mode. Helium (5.0) was used as a carrier gas with a flow rate of 1.22 ml/min. The column oven was programmed as follows: starting at 50°C, the temperature was increased at a rate of 10°C/min to 300°C, which was held for 10 min. Mass spectra were obtained by electron ionization (EI; 70 eV). The temperatures of the ion source and interface were set to 250°C. Data were recorded from *m/z* 50 to 500 with a rate of 10 scans/s. For comparison of retention times and fragmentation patterns, a bacterial acid methyl ester standard solution (BAME) (47080-U; Sigma-Aldrich, Steinheim, Germany) was used (10-fold diluted with methyl *tert*‐butyl ether).

### Computational methods.

The evolutionary history was inferred using the neighbor-joining method (62). Evolutionary analyses were conducted in MEGA7 (63).

### Accession numbers.

The corrected sequence of the *rhlA* gene in *P. ananatis* LMG 20103 and the sequence containing PhaG in *P. desmolyticum* were deposited under the GenBank accession numbers MF671909 and MG099922, respectively. The codon-optimized *rhlA* homologs for the construction of pHAL and pPLY are accessible under MN369027 and MN369028.
